# Research on the Guidance of Youth Labor Education Based on the “Combination of Education and Production Labor” Program Based on the Deep Learning Model

**DOI:** 10.1155/2022/2576559

**Published:** 2022-10-11

**Authors:** Linyu Xiao, Xiaoyi Liao

**Affiliations:** ^1^Hunan Agricultural University, Institute of Marxism, Changsha 410128, Hunan, China; ^2^Hunan Agricultural University, College of Horticulture, Changsha 410128, Hunan, China

## Abstract

At present, there is a lack of research on Marx's idea of “combining education and productive labor” and its guiding significance for youth labor education, and no effective teaching model has been formed. In response to this problem, this study proposes a semi-supervised deep learning model based on u-wordMixup (SD-uwM). When there is a shortage of labeled samples, semi-supervised learning uses a large number of unlabeled samples to solve the problem of labeling bottlenecks. However, since the unlabeled samples and labeled samples come from different fields, there may be quality problems in the unlabeled samples, which makes the generalization ability of the model worse., resulting in a decrease in classification accuracy. The model uses the u-wordMixup method to perform data augmentation on unlabeled samples. Under the constraints of supervised cross-entropy and unsupervised consistency loss, it can improve the quality of unlabeled samples and reduce overfitting. The comparative experimental results on the AGNews, THUCNews, and 20Newsgroups data sets show that the proposed method can improve the generalization ability of the model and also effectively improve the time performance. The study found that the SD-uwM model uses the u-wordMixup method to enhance the unlabeled samples and combines the idea of the Mean Teacher model, which can significantly improve the text classification performance. The SD-uwM model can improve the generalization ability and time performance of the model, respectively, 86.4 ± 1.3 and 90.5 ± 1.3. Therefore, the use of SD-uwM in Marx's program is of great practical significance for the guidance process of youth labor education.

## 1. Introduction

From the standpoint of the proletariat, Marx opposed the exploitation and oppression of workers by the bourgeoisie, combined with the actual development of the modern machinery industry to construct a theoretical system combining productive labor and education, and demonstrated its inevitability in the process of social development [1]. This theory also had a profound impact on Marx's educational thought and provided important guidance on how the proletariat should carry out educational activities. In our country, it is very necessary to excavate the connotation of Marx's thought of “combining education with productive labor,” which has theoretical value and practical significance. This thought of Marx can guide our country to educate and cultivate laborers with professional quality and promote the improvement of productivity, thereby promoting social development [2]. Since the 18th National Congress of the Communist Party of China, General Secretary Xi Jinping has delivered many important speeches on the topic of labor based on Marx's discussion on labor. He highly recognized the important position of labor in socialist countries, and also clarified the importance of cultivating young people's correct labor values and loving physical labor in the new era. General Secretary Xi Jinping clearly stated at the 2018 National Education Conference: “We must educate and guide students to advocate and respect labor.” [3] In today's society, we must attach importance to labor education and give full play to the educating function of physical labor and use Marxist labor Ideology arm the minds of young people, guide them to love labor, and train young people into laborers who meet the needs of social development in practice, so that they can better serve my country's modernization drive. At the same time, the “Opinions on Comprehensively Strengthening Labor Education in Colleges and Elementary Schools in the New Era” issued by my country pointed out that labor education is an indispensable part and an important part of education in the education system of socialist countries. We must pay attention to the unique educational value of labor and strengthen modern labor education [4]. Through classroom teaching, labor practice, etc., effective measures are taken to change the attitude of young people towards labor and to curb the phenomena of extravagance, lack of progress, and greed for pleasure in the campus. In this context, it is more necessary to study Marx's thought of “combining education and productive labor” and its guiding significance for youth labor education.

The famous Russian educator Ushinsky believes that education is an important hand to guide students to respect and love labor, and it is also an indispensable part of cultivating students' labor habits [5, 6] Lenin attached great importance to the combination of education and productive labor. He inherited and developed Marx's idea and pointed out that the ideal realization of the future society cannot be separated from the combination of education and productive labor of the new generation. In a socialist country, labor courses should be an indispensable course in school education. Students can master labor skills by participating in voluntary labor and combine practice and theory to help themselves better master scientific knowledge and become socialist laborers [7]. Makarenko believes that labor has an important impact on a person's future development. Individual labor can improve one's living standard and enhance happiness; in collective labor, people help each other and can establish good interpersonal relationships [8]. In the book “Dedicate the whole heart to the child,” Sukhomlinsky said that the beauty of human beings is the brightest in labor, so an important task of education is to make the child's surroundings full of the natural world and through labor a world created and built. Labor has the function of aesthetic education, and schools should make labor a need for students' spiritual life and become a powerful educational force on this basis. Lenin, Makarenko, and Sukhomlinsky all stood from the perspective of the proletariat and believed that the ultimate goal of labor education was to train laborers for a communist society. The discussion of this topic by British scholars can be traced back to the early utopian socialist Thomas More who required children to learn agricultural knowledge in school, go to work in the fields, and required every youth to learn at least one kind of handicraft. Owen also mentioned that people will form their own characters during labor, so labor education should be paid attention to in childhood [9]. Locke believes that labor can make people physically and mentally healthy and overcome many vices. Students can choose one of crafts, gardening, or agriculture to practice [10]. German educator Comenius said in “The Great Teaching Theory” that when realizing the ideal of education through labor, attention should be paid to adapting to the natural order, adapting to human nature and age characteristics. Pestalozzi, a Swiss educator, was the first educator to put the idea of combining education and labor into practice in the history of Western education. He believed that this method could coordinate and develop people's abilities in all aspects and advocated the integration of education and labor [11].

Through the consistency training framework [12], based on the wordMixup data enhancement method, this paper proposes a u-wordMixup (unlabeled sample word mixture) data enhancement method for unlabeled samples. Under the constraint of loss, the enhancement generates high-quality additional training samples and reduces overfitting. Based on the u-wordMixup method, a new semi-supervised deep learning model (SD-uwM) is proposed using the Mean Teacher model for consistent training. The u-wordMixup data augmentation method enhances unlabeled samples with the goal of reducing unsupervised consistency loss, constrains the quality of unlabeled training samples, and reduces model overfitting. The objective loss function combines supervised cross-entropy loss and unsupervised consistency loss and uses the MeanTeacher method for consistency training to improve the generalization ability of the model. Optimize the key words of the “Combination of Education and Productive Labor” program to achieve the optimal application of youth education.

## 2. Principles and Methods

### 2.1. wordMixup Data Enhancement

wordMixup is a data enhancement method for labeled samples. The idea is to interpolate the word embedding vectors of two samples to generate a new sample word embedding matrix as an enhanced sample [13]. Given a pair of labeled samples (*x*_*i*_, *y*_*i*_) and (*x*_*j*_, *y*_*j*_), perform word embedding to get (*x*_*i*_, *y*_*i*_) and (*x*_*j*_, *y*_*j*_), where *x*_*i*_ ∈ RN × *d*, *x*_*j*_ ∈ RN × *d* is the word embedding matrix of text *x*_*i*_ and *x*_*j*_, *N* is the number of words, *d* is the word vector dimension, and *y*_*i*_ and *y*_*j*_ are the corresponding class labels. Then perform interpolation according to formulas (1) and (2) to obtain a new sample (*x*_*ij*_, *y*_*ij*_), where *x*_*ij*_ is the word embedding matrix of the enhanced sample, and *y*_*ij*_ is its class label.

(1) $$\begin{array}{c} {\tilde{x}}_{ij}^{k}=\lambda x_{i}^{k}+\left(1-\lambda \right)x_{j}^{k},\;k=1\ldots N, \end{array}$$

(2) $$\begin{array}{c} {\tilde{y}}_{ij}=\lambda y_{i}+\left(1-\lambda \right)y_{j}. \end{array}$$

Among them, *x*_*i*_^*k*^ and *x*_*j*_^*k*^ represent the word vector of the *k*th word in the text *x*_i_ and *x*_*j*_, respectively, *λ* ∈ [0, 1] is the interpolation weight factor, and *x*_*ij*_^*k*^ is the word vector of the *k*th word of the new sample generated by interpolation. Perform word vector interpolation on each word in (*x*_*i*_, *y*_*i*_) and (*x*_*j*_, *y*_*j*_) one-to-one to obtain the embedding matrix *x*_*ij*_ of the new sample, where *y*_*ij*_ is the class label of *x*_*ij*_, and (*x*_*ij*_, *y*_*ij*_) is the enhanced additional training samples.

The wordMixup method achieves good results in supervised text classification. But unlabeled in semi-supervised learning has no labels, how to generate pseudo labels for its interpolation? To this end, based on wordMixup, this paper proposes an improved data enhancement method u-wordMixup for unlabeled samples, which will be introduced in the following chapters [14].

### 2.2. SD-uwM Semi-Supervised Deep Learning Model

#### 2.2.1. SD-uwM Model

**Figure 1:**
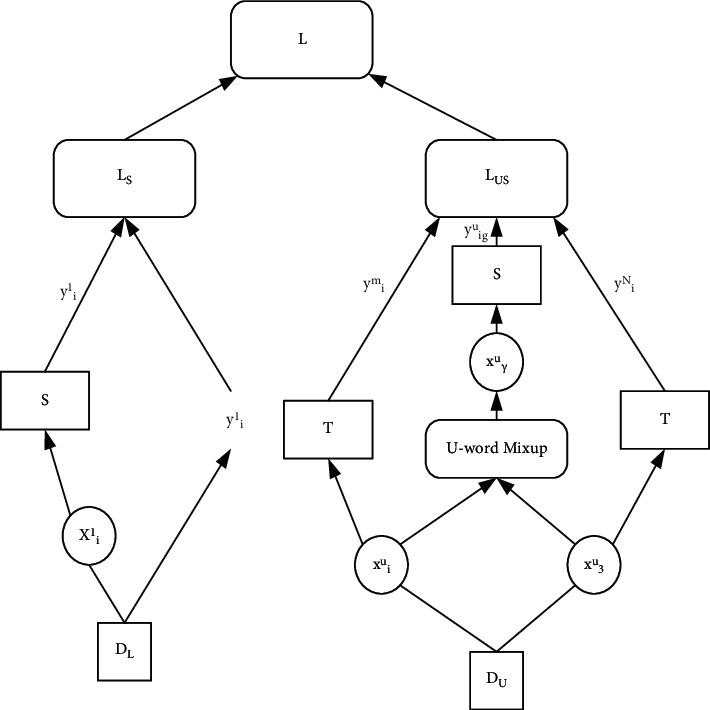
SD-uwM model.

The framework of the semi-supervised deep learning model (SD-uwM) is shown in Figure 1 [15]. It uses the idea of the Mean Teacher model to construct a teacher model *T* and a student model *S*, using labeled samples and unlabeled samples, based on supervised cross-entropy loss and unsupervised consistency loss objective functions for semi-supervised deep learning [16].

As shown in Figure 1, *DL*={(*xli*, *yli*)}Nl i=1 represents the labeled training sample set, *DU*   = {(xui )}Nui=1 represents the unlabeled training sample set, Nl represents the labeled sample set number, and Nu represents the number of unlabeled samples. *T* is the teacher model, *S* is the student model, and *T* and *S* have the same network structure. LS stands for supervised loss, LUS stands for unsupervised loss, and *L* stands for consistency objective loss function [17].

SD-uwM trains and learns simultaneously on labeled samples and unlabeled samples based on a consistent target loss function *L* [18]. As shown in the left half of Figure 1, the student model *S* computes a supervised cross-entropy loss LS on DL. At the same time, as shown in the right half of Figure 1, the student model *S* uses the u-wordMixup method to enhance the unlabeled samples. According to the prediction of the enhanced samples by the student model *S* and the prediction of the unlabeled samples by the teacher model *T*, the calculation is unsupervised and consistent. The performance loss LUS, LS and LUS together constitute the consistency target loss function *L* of the SD-uwM model. After many iterations, the SD-uwM model is trained to output the parameters of the student model *S* as the parameters of the final classification model [19].

#### 2.2.2. u-wordMixup Data Enhancement

Using the Mean Teacher model idea and consistency training [20], the target loss function *L* of SD-uwM takes into account both supervised cross-entropy loss and unsupervised consistency loss and is defined as shown in

(3) $$\begin{array}{c} L=L_{S}+\beta L_{US}, \end{array}$$

where LS is the supervised cross-entropy loss on the labeled samples DL, LUS is the unsupervised consistency loss on the unlabeled samples DU, and *β* is the scale coefficient. LS is the supervised loss of the student model *S* on the labeled sample set DL, which is calculated as

(4) $$\begin{array}{c} L_{S}=E_{x_{i}^{l},y_{i}^{l}\in D_{L}}\left[-y_{i}^{l}\log\;p_{\theta \prime }\left(x_{i}^{l}\right)\right]. \end{array}$$

Among them, *y*_*i*_^*l*^ is the true label of the labeled sample *x*_*i*_^*l*^, *θ*′ represents the parameter of the student model S, and *p*_*θ*_′(*x*_*i*_^*l*^) represents the predicted pseudo-label of the sample *x*_*i*_^*l*^ by the student model *S*, that is, *y*_*i*_^*l*^.

**Figure 2:**
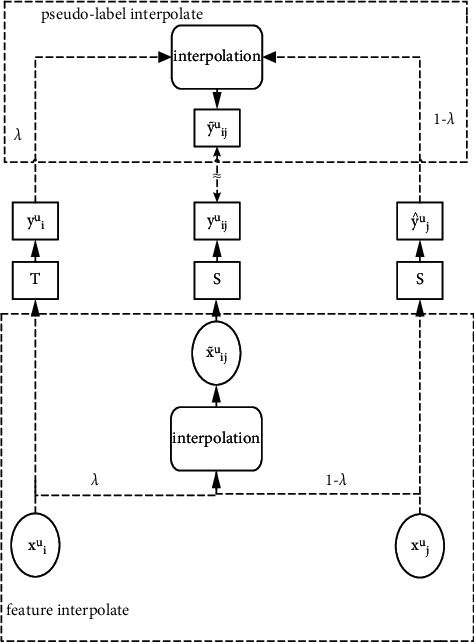
u-wordMixup method.

Based on wordMixup, a u-wordMixup data augmentation method for unlabeled samples is proposed as part of the SD-uwM model [21]. Different from the wordMixup method, the interpolation operation object of the u-wordMixup method has no real class label. The u-wordMixup method is shown in Figure 2 [22].

As shown in Figure 2, *x*_*i*_^*u*^ and *x*_*j*_^*u*^ represent two unlabeled samples, where *x*_*i*_^*u*^ ∈ RN × *d*, *x*_*j*_^*u*^ ∈ RN × *d*, *N* is the number of words, and *d* is the word vector dimension. The feature interpolation of the word vector is performed for each word in *x*_*i*_^*u*^ and *x*_*j*_^*u*^ one-to-one, and the embedding matrix ${\tilde{x}}_{ij}^{u}$ of a new unlabeled sample is obtained as an additional training sample [23]. The teacher model *T* predicts the unlabeled samples *x*_*i*_^*u*^ and *x*_*j*_^*u*^ to generate pseudo-labels ${\hat{y}}_{i}^{u}$ and ${\hat{y}}_{j}^{u}$ and performs pseudo-label interpolation on${\hat{y}}_{i}^{u}$ and ${\hat{y}}_{j}^{u}$ to obtain ${\tilde{y}}_{ij}^{u}$, which is used as the pseudo-label of the enhanced sample ${\tilde{x}}_{ij}^{u}$. Then the student model S predicts the enhanced sample ${\tilde{x}}_{ij}^{u}$ to get the predicted label ${\hat{y}}_{ij}^{u}$. Among them, the calculation of feature interpolation and pseudo-label interpolation are as follows:

(5) $$\begin{array}{c} {\tilde{x}}_{ij}^{uk}=q_{\lambda }\left(x_{i}^{u},x_{j}^{u}\right)=\lambda x_{i}^{uk}+\left(1-\lambda \right)x_{j}^{uk},\;k=1\ldots N, \end{array}$$

(6) $$\begin{array}{c} {\tilde{y}}_{ij}^{u}=q_{\lambda }\left({\hat{y}}_{i}^{u},{\hat{y}}_{j}^{u}\right)=\lambda {\hat{y}}_{i}^{u}+\left(1-\lambda \right){\hat{y}}_{j}^{u}. \end{array}$$

Among them, *qλ* (*x*_*i*_^*u*^, *x*_*j*_^*u*^) represents the u-wordMixup data enhancement transformation, *x*_*ki*_^*u*^ and *x*_*kj*_^*u*^ represent the word vector of the *k*th word of the samples *x*_*i*_^*u*^ and *x*_*j*_^*u*^, respectively, *λ* ∈ [0, 1] is the interpolation weight factor, and ${\tilde{x}}_{kij}^{u}$ is the interpolation. The word vector of the *k*th word of the generated augmented sample. ${\hat{y}}_{i}^{u}$ is the predicted pseudo-label of *x*_*i*_^*u*^ by the teacher model *T*, ${\hat{y}}_{j}^{u}$ is the predicted pseudo-label of *x*_*i*_^*u*^ by the teacher model *T*, and ${\tilde{y}}_{ij}^{u}$ is the pseudo-label generated by interpolation, that is, the pseudo-label of ${\tilde{x}}_{ij}^{u}$.

Based on consistent training, the pseudo-label predicted by the student model S for the enhanced unlabeled sample ${\tilde{x}}_{ij}^{u}$ is ${\hat{y}}_{ij}^{u}$, which should be as consistent as possible with the pseudo-label ${\tilde{y}}_{ij}^{u}$ generated by interpolation, that is, ${\tilde{y}}^{u}{}_{ij}\approx {\hat{y}}^{u}{}_{ij}$, and ideally the two are equal. Therefore, the unsupervised consistency loss LUS is computed as

(7) $$\begin{array}{c} L_{US}=E_{x_{i}^{u},x_{j}^{u}\in D_{U}}E_{{\tilde{x}}_{ij}^{u}∼q_{\lambda }\left(x_{i}^{u},x_{j}^{u}\right),\lambda \in \left[0,1\right]}\left[-\left(\lambda p_{\theta }\left(x_{i}^{u}\right)+\left(1-\lambda \right)p_{\theta }\left(x_{j}^{u}\right)\right)\log\;p_{\theta^{\prime }}\left({\tilde{x}}_{ij}^{u}\right)\right], \end{array}$$

where *θ* denotes the parameters of the teacher model *T*, *θ*′ denotes the parameters of the student model *S*, and *θ* is the moving average of *θ*′. *p*_*θ*_(*x*_*i*_^*u*^) represents the predicted pseudo-label of the sample *x*_*i*_^*u*^ by the teacher model *T*, *p*_*θ*_(*x*_*j*_^*u*^) represents the predicted pseudo-label of the sample *x*_*j*_^*u*^ by the teacher model *T*, and *p*_*θ*′_(${\tilde{x}}_{ij}^{u}$) represents the prediction of the newly generated sample ${\tilde{x}}_{ij}^{u}$ by the student model S Pseudo tags. *λ* ∈ [0, 1] is the interpolation weight factor, and *qλ*(*x*_*i*_^*u*^, *x*_*j*_^*u*^) represents the u-wordMixup data augmentation transformation [24].

The deep semi-supervised learning SD-uwM model uses the u-wordMixup method to enhance the unlabeled samples. The unsupervised consistency loss LUS aims to reduce the consistency loss and constrain the quality of the enhanced unlabeled samples. Combined with the Mean Teacher model, the teacher model *T* and the student model S are constructed, and the labeled samples and unlabeled samples are trained. The weighted summation of LS and LUS is used as the objective function *L* of the model SD-uwM [25].

#### 2.2.3. SD-uwM Model Application

The algorithm description of SD-uwM is shown in Algorithm 1 [26]. The objective function *L* of the algorithm takes into account the supervised cross-entropy loss LS and the unsupervised consistency loss LUS, and the constraint enhancement generates unlabeled training samples [27]. In each iteration, according to the objective function *L*, the parameter *θ*′ of the student model S is optimized. After many iterations, the parameter *θ*′ of the optimal student model *S* is finally obtained.

## 3. Test Analysis

### 3.1. Data Set

This article selects three data sets: AGNews, 20Newsgroups and THUCNews. AGNews selects four categories of “world,” “politics,” “education” and “labor,” and 20Newsgroups selects “alt.atheism,” “soc.religion.Christian,” “comp.graphics,” and “sci.med” 4 categories, THUCNews selects 4 categories of “Education,” “Labor,” “Program,” and “Technology.” The scaling factor *β* is set to 1 in Algorithm 1.

### 3.2. Analysis of Experimental Results

The comparison method in the experiment is as follows:

1. SD-uwM: the semi-supervised deep learning model based on u-wordMixup data augmentation proposed in this paper
2. wM-SL: a supervised text classification method based on wordMixup data augmentation method in the literature
3. SL: supervised text classification method without data augmentation
4. Mean Teacher: semi-supervised method applied to image classification in the literature, modified for text classification tasks

#### 3.2.1. Comparison of Target Loss between SD-uwM and Mean Teacher

**Figure 3:**
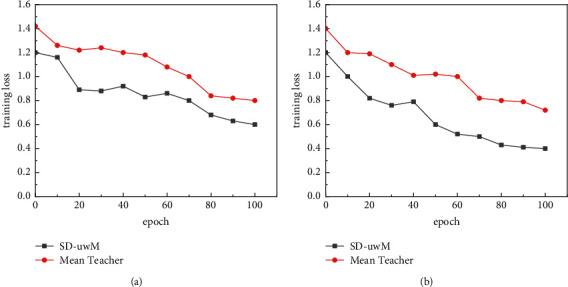
Comparison of training loss between SD-uwM and Mean Teacher when TextCNN is selected: (a) Nl = 200, Nu = 3000 (THUCNews data set); (b) Nl = 300, Nu = 5000 (AGNews data set).

In order to verify the effectiveness of the u-wordMixup method, a comparative experiment was conducted on SD-uwM and Mean Teacher models, and the training loss changes are shown in Figure 3.

As can be seen from Figure 3, compared to the Mean Teacher model, the SD-uwM model using the u-wordMixup data augmentation method has lower training loss. This is because the SD-uwM model objective loss function is more realistic, in which LUS is combined with the u-wordMixup method, aiming to reduce the unsupervised consistency loss, which can improve the quality of unlabeled samples, thereby improving the performance of the model.

#### 3.2.2. Classification Comparison between SD-uwM Model and Other Methods

*(1) Comparison of the Accuracy of SD-uwM Model with Other Methods*. SD-uwM model and Mean Teacher, wM-SL, SL model on AGNews(Nl = 300, Nu = 5000), THUCNews(Nl = 300, Nu = 5000), and 20 Newsgroups (Nl = 200, Nu = 2000) The comparative experimental results are shown in Table 1.

As can be seen from Table 1, on the three data sets, regardless of whether the network structure is LSTM or TextCNN, the classification accuracy of the SD-uwM model is better than that of the SL, wM-SL, and Mean Teacher models, up to 90.4. The SL model is a supervised learning method, which requires a large number of labeled samples to achieve better performance. The wM-SL model only enhances the labeled samples, and the Mean Teacher model does not use the u-wordMixup method to enhance the samples. The SD-uwM model uses the u-wordMixup method to enhance the data of unlabeled samples and uses the unsupervised consistency loss constraint to improve the generalization ability of the model.

**Figure 4:**
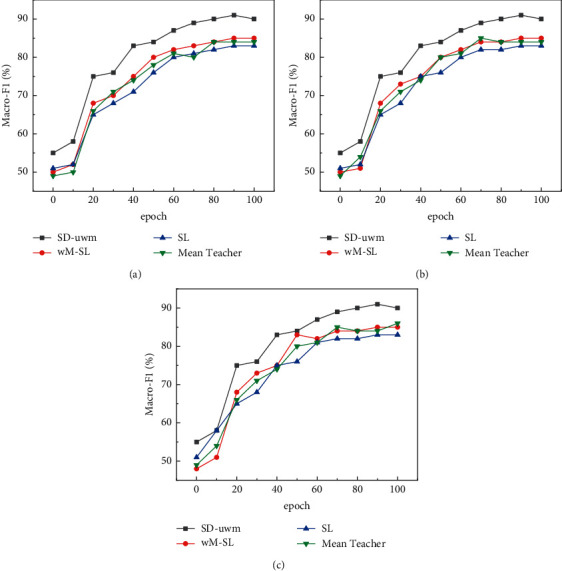
Comparison of macro-F1 value of each model with iteration times on three data sets using LSTM: (a) AGNews data set; (b) THUCNews data set; (c) 20Newsgroups data set.

**Figure 5:**
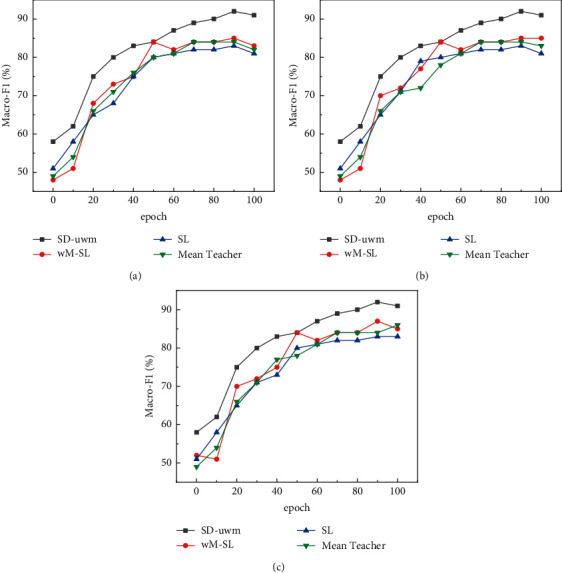
Comparison of the macro-F1 value of each model with the number of iterations on three data sets using TextCNN: (a) AGNews data set; (b) THUCNews data set; (c) 20 Newsgroups data set.

*(2) Comparison of Classification Performance of SD-uwM Model with Increasing Iterations*. The number of labeled samples and the number of unlabeled samples are fixed. As the number of iterations increases, the changes in the Macro-F1 value of the SD-uwM model and the SL, wM-SL, and Mean Teacher models are compared and analyzed. On AGNews (Nl = 300, Nu = 5000), THUCNews (Nl = 300, Nu = 5000), and 20Newsgroups (Nl = 200, Nu = 2000), the experimental results are shown in Figures 4 and 5.

It can be seen from Figures 4 and 5 that with the increase of the number of iterations, although the indicators of the SL, wM-SL, Mean Teacher, and SD-uwM models generally show an upward trend and converge to a certain upper limit, the SD-uwM model classification. The performance is significantly better than SL, wM-SL, and Mean Teacher models. As shown in Figure 5(a), using LSTM on AGNews, compared with Mean Teacher, wM-SL, and SL, the Macro-F1 of SD-uwM reaches 90.3%, which is increased by 8%, 9.9%, and 14.5%, respectively. This is because SD-uwM uses the u-wordMixup method to target unsupervised consistency loss for unlabeled sample enhancement, which can reduce overfitting and improve classification performance.

As can be seen from the above Figure 5, in order to verify the influence of unlabeled samples on the SD-uwM model, the number of labeled samples Nl = 300 is fixed on AGNews and THUCNews, the number of labeled samples Nl = 200 is fixed on 20Newsgroups, and the number of unlabeled samples is constantly increasing. Compare the classification results of SD-uwM model with SL, wM-SL, and Mean Teacher models. It can be seen that the indicators of SD-uwM and Mean Teacher show an upward trend with the increase of unlabeled samples, but the classification results of SD-uwM are significantly better than that of Mean Teacher, wM-SL, and SL. Using LSTM on THUCNews, compared with Mean Teacher, wM-SL, and SL, the Macro-F1 of SD-uwM has reached 91.4%, an increase of 5.3%, 8.2%, and 13.9%, respectively. It can be seen that since the SD-uwM model uses the u-wordMixup method to enhance the unlabeled samples and combines the idea of the Mean Teacher model, it can improve the text classification performance.

#### 3.2.3. Time Performance Analysis of the SD-uwM Model

When selecting unlabeled samples, the usual semi-supervised learning algorithm needs to calculate the similarity matrix between the unlabeled samples and the labeled samples, which will increase the time complexity. The SD-uwM model in this paper is random sampling, and there is no need to calculate the similarity between the two samples. The temporal performance comparison between SD-uwM model and typical semi-supervised learning method Co-training is shown in Table 2.

As can be seen from Table 2, the classification accuracy of the SD-uwM model is 86.4% ± 1.3 and 90.5% ± 1.3 in the two different data sets, respectively, and the classification accuracy of the SD-uwM model is significantly higher than that of the co-training model. The classification accuracy was 83.3% ± 1.2 and 88.4% ± 1.2, respectively. At the same time, the time performance of SD-uwM model is significantly better than that of co-training, in which SD-uwM model time is basically maintained at 0.01–0.02 s, while co-training model has exceeded 30 s. The reason is that when selecting unlabeled training samples, the SD-uwM model is random sampling, and the time complexity is O(1), while the co-training method needs to calculate the sample similarity matrix, and the time complexity is O(Nl ∗ Nu).

## 4. Conclusion

It is extremely necessary to study Marx's thought of “combining education with productive labor” and its guiding significance to youth labor education. This paper proposes a u-wordMixup method for the data augmentation of unlabeled samples and combines the consistent training framework and the Mean Teacher method to propose a semi-supervised deep learning model SD-uwM. The model uses the u-wordMixup method to enhance the data of unlabeled samples and takes into account the supervised cross-entropy loss and unsupervised consistency loss to construct a new objective function, so as to realize the teaching optimization of the combination of education and production labor. The findings of the study show that the experimental days are as follows:

1. On the three data sets, regardless of whether the network structure is LSTM or TextCNN, the classification accuracy of the SD-uwM model is better than that of the SL, wM-SL, and Mean Teacher models
2. SD-uwM model can improve the generalization ability and time performance of the model, which are 86.4 ± 1.3 and 90.5 ± 1.3, respectively
3. Since the SD-uwM model uses the u-wordMixup method to enhance the unlabeled samples and combines the idea of the Mean Teacher model, it can improve the performance of text classification

## Figures and Tables

**Table 1:** Classification comparison of four models on three classification results.

Model	Network structure	Accuracy		
		AGNews	THUCNews	20Newsgroups
SL	LSTM	75.4 ± 1.1	77.5 ± 1.3	71.5 ± 1.3
wM-SL		80.4 ± 1.3	83.2 ± 1.2	75.4 ± 1.3
Mean Teacher		82.1 ± 1.3	86.1 ± 1.3	77.5 ± 1.1
SD-uwM		90.4 ± 1.2	91.4 ± 1.3	85.4 ± 1.2
SL	TextCNN	76.4 ± 1.2	78.4 ± 1.4	71.2 ± 1.2
wM-SL		80.5 ± 1.2	84.5 ± 1.3	75.3 ± 1.2
Mean Teacher		83.6 ± 1.1	86.1 ± 1.5	78.1 ± 1.3
SD-uwM		91.2 ± 1.3	92.2 ± 1.3	86.2 ± 1.1

**Table 2:** Time performance comparison of SN-uwM and co-training.

Dataset	Model	Accuracy (%)	Time (s)	Ratio of train time (SD-uwM/co-training)
20 Newsgroups	SD-uwM	86.4 ± 1.3	0.01	1/3000
Nl = 200, Nu = 2000	Co-training	83.3 ± 1.2	30	
THUCNews	SD-uwM	90.5 ± 1.3	0.02	1/2200
Nl = 300, Nu = 4000	Co-training	88.4 ± 1.2	44	

## Data Availability

The data set can be accessed upon request.
